# Curcumin Administered in Combination with Glu-GNPs Induces Radiosensitivity in Transplanted Tumor MDA-MB-231-luc Cells in Nude Mice

**DOI:** 10.1155/2021/9262453

**Published:** 2021-11-16

**Authors:** Mengjie Li, Ling Lin, Tingting Guo, Yujian Wu, Jiayi Lin, Yuanyuan Liu, Ke Yang, Chenxia Hu

**Affiliations:** ^1^School of Pharmaceutical Science, Guangzhou University of Chinese Medicine, 510006 Guangzhou, Guangdong, China; ^2^Pharmacy Department, Guilin Municipal Hospital of Traditional Chinese Medicine, Guilin, Guangxi, China; ^3^International Institute for Traditional Chinese Medicine, Guangzhou University of Chinese Medicine, 510006 Guangzhou, Guangdong, China

## Abstract

Curcumin is a type of plant polyphenol extracted from *Curcuma longa* L. rhizome, which demonstrates antitumor activity in breast cancer cells *in vitro*. To investigate the combined effect and possible mechanism of curcumin and glucose-gold nanoparticles (Glu-GNPs), the radiosensitivity of breast carcinoma xenografts was assessed in nude mice. *MDA-MB-231 cells labeled with* firefly *luciferase* were inoculated into the mammary fatty pads of nude mice to establish a transplantation tumor model of human breast cancer. The tumor-bearing mice were treated with different drugs (curcumin, Glu-GNPs, and cisplatin) for 3 weeks prior to radiotherapy. The body weights and tumor volumes of the mice were measured in regular intervals. Tumor bioluminescence intensity was determined in real-time using an *in vivo* bioluminescence imaging system to monitor tumor growth. Transplanted tumor tissue samples were taken for hematoxylin and eosin (HE) staining, and the expression of VEGF, HSP90, HIF-1*α*, and MMP9 was evaluated via reverse transcription-quantitative PCR or immunohistochemistry. The results revealed that the breast tumor-bearing nude mouse model was successfully established, as evidenced by a stable expression of luciferase. Curcumin inhibited the growth of tumors without causing significant weight loss in mice. Furthermore, additive inhibition was demonstrated when curcumin was administered in combination with Glu-GNPs and irradiation. Tumor bioluminescence intensity was decreased in the model group following curcumin, Glu-GNPs, and irradiation treatment. HE staining demonstrated that transplanted tumors were malignant, with necrotic tissue exhibited centrally. It was concluded that curcumin administered in combination with Glu-GNPs and X-ray irradiation could reduce the protein expression of VEGF, HSP90, HIF-1*α*, and MMP9 in tumor tissue when compared with the model group. Curcumin and Glu-GNPs administered with X-ray irradiation significantly inhibited tumor growth and induced radiosensitivity, which may be associated with the inhibition of angiogenesis in tumor tissue.

## 1. Introduction

According to the data published by the International Cancer Research Institute in 2018, breast cancer has become the second leading cause of cancer-related deaths and the first leading cause of cancer-related deaths in women, worldwide [1]. It is also expected that the morbidity and mortality of breast cancer will significantly increase in the next few years [2]. Current treatment of breast cancer primarily includes surgery, chemotherapy, and radiotherapy [3, 4]. Despite the great progress that has been made in the treatment of early breast cancer, there is no effective strategy to treat breast cancer with chemoradiotherapeutic resistance.

Gold nanoparticles (GNPs), a type of nanomaterial, have been approved for use in clinical trials by the US FDA. Their use in tumor diagnosis and radiotherapy has therefore gained increasing interest [5]. It has been reported that when X-ray irradiation was applied to GNPs, the ionizing ability of the radiation applied to tumor cells was enhanced, which promoted tumor cell damage and apoptosis [6–9]. It has also been demonstrated that glucose tagging may be a suitable method for promoting GNP uptake in tumor cells, as this cell type can uptake more glucose compared with normal cells. A previous study revealed that glucose-gold nanoparticles (Glu-GNPs) were further assimilated in MCF-7 adherent cells compared with GNPs, increasing the radiosensitivity of breast cancer cells *in vitro* [10]. Moreover, it was previously demonstrated that these nanometal particles administered to mice enhanced the tumor cell killing effect without increasing damage to the surrounding normal tissue, thereby reducing the adverse effects of radiotherapy [11, 12].

Curcumin, a plant polyphenol extracted from the rhizome of *Curcuma longa*, is the main ingredient of curry and has a high efficiency to anticancer, safety, and low toxicity [13]. It has been demonstrated both *in vitro* and *in vivo* that curcumin serves killing and therapeutic effects in different types of cancer, including lung [14], gastric [15], and breast cancer [16]. Hu et al. [17] revealed that curcumin inhibited the proliferation and induced the apoptosis of various breast cancer cells, including MDA-MB-231, MCF-7, MDA-MB-468, and T47D. It has also been determined that curcumin inhibited the proliferation, invasion, migration, and epithelial to mesenchymal transition of adherent and stem-like breast cancer cells *in vitro* [18]. These results suggested that curcumin may have important significance for the treatment of patients with breast cancer. However, whether curcumin and Glu-GNPs administered alone or in combination can enhance radiosensitivity in breast cancer cells *in vivo* is yet to be elucidated.

In recent years, bioluminescence imaging systems have become an efficient method for use in animal research. It can monitor the growth and metastasis of tumors in real time. In addition, it can be accurately detected *in vivo* without harming mice [19]. Zhang et al. [20] revealed that the results of *in vivo* imaging and pathological detection are consistent, and when compared with hematoxylin and eosin (HE) staining, the growth and metastasis of breast cancer can be more directly and clearly observed using *in vivo* imaging systems. Therefore, *in vivo* imaging methods are applied to assess transplanted tumors in mice. Compared with traditional measurement methods, *in vivo* imaging encompasses scientific systems to measure tumor growth more accurately, which greatly reduces the error of artificial measurement.

The current study used the MDA-MB-231-luc cell line to establish a subcutaneous transplant tumor model. Tumor-bearing mice were treated with curcumin, Glu-GNPs, and irradiation alone or in combination for 3 weeks. The tumor volume and body weight of tumor-bearing mice were measured regularly, and tumor growth was monitored using an *in vivo* imaging system. The results demonstrated that curcumin inhibited tumor growth in nude mice and reduced tumor fluorescence intensity but did not demonstrate a marked effect on murine body weight. Further results suggested that curcumin and Glu-GNPs administered alongside X-ray irradiation downregulated the mRNA and protein expression of VEGF and heat shock protein (HSP) 90. These data revealed that curcumin may serve as a potential drug for breast cancer treatment.

## 2. Materials and Methods

### 2.1. Reagents and Antibodies

Curcumin (Sigma-Aldrich; Merck KGaA; purity ≥ 80%) was dissolved in corn oil with 2% DMSO. Glu-GNPs were obtained from Beijing Dk Nano Technology Co., Ltd., at a concentration of 1 mg/ml. Cisplatin (Sigma-Aldrich; Merck KGaA) was included as a positive control and dissolved in normal saline. The following primary antibodies were obtained from Cell Signaling Technology, Inc.: *β*-actin (cat. no. #4970) and HSP90 (cat. no. #4877). Additionally, antibodies against VEGF (cat. no. ab52917) and antibodies against HIF-1*α* (cat. no. ab113642) were purchased from Abcam. MMP9 (cat. no. PB9669) and goat anti-rabbit and goat anti-mouse secondary antibodies were obtained from Boster Biological Technology Ltd.

### 2.2. Cell Culture

Human breast cancer *MDA-MB-231 cells labeled with* firefly *luciferase* (MDA-MB-231-luc) were obtained from RUANTUO BIO (http://www.ruantuobio.com) and cultured in Modified Eagle's Medium supplemented with 10% FBS, 100 U/ml penicillin, and 100 *μ*g/ml streptomycin (Gibco; Thermo Fisher Scientific, Inc.) at 37°C in a humidified incubator with 5% CO_2_ (Thermo Fisher Scientific, Inc.). Cells were passaged at a density of 2.0 × 10^5^ cells in a 25 cm^2^ flask or cryopreserved at a density of 2.0 × 10^6^/ml.

### 2.3. Detection of MDA-MB-231-luc Bioluminescence

The bioluminescence intensity of MDA-MB-231-luc cells was measured using an *in vivo* imaging system (Night OWLIILB 983 Imaging System, Berthold, Germany). Cells were inoculated at different densities (1.25 × 10^4^, 2.5 × 10^4^, and 5 × 10^4^) onto a black opaque 96-well plate and incubated for 4 h to induce cell attachment. According to the protocol of the Firefly-Luciferase Assay Kit, luciferase substrate was added to cells following treatment with lysate. The luminescence of samples was detected using the *in vivo* imaging system immediately after treatment.

### 2.4. Establishment of Subcutaneous Transplanted Tumors in Nude Mice

Female *BALB/c-nu/nu* mice aged 4-6 weeks (Certificate No. 44005800008110) were purchased from Guangzhou University of Chinese Medicine, and the animal experiment was approved by the Animal Ethics Committee of Guangzhou University of Chinese Medicine (No.00199983). All nude mice were housed at 26-28°C and 40-60% humidity and provided for standard mouse chows and pure water in SPF environment, and the indoor air is ventilated 10-15 times per hour. Nude mice were reared in a spacious space with 10 hours of light and 14 hours of darkness every day. The nude mice were raised adaptively for 7 days, after which they were randomly divided into the following groups (*n* = 5 per group): control, model, cisplatin (Cis), curcumin (Cur), irradiation (IR), curcumin+Glu-GNP group (Cur+Glu-GNPs), and curcumin+Glu-GNPs+irradiation (Cur+Glu-GNPs+IR). All nude mice except for those of the control group were inoculated with 0.1 ml cell suspension containing 1.5 × 10^7^/ml MDA-MB-231-luc cells in PBS and Matrigel (BD Biosciences; 1 : 1) into the second pair of subcutaneous mammary glands on the left-hand side. Nude mice in the control group were inoculated with a 0.1 ml suspension of PBS and Matrigel (1 : 1). After 3-7 days, tumors were formed at the site of inoculation in all mice, except those of the control group. When the diameter of transplanted tumor was >5 mm, the model was successfully established. The tumor volume was measured every 3 days, and the body weight of mice was assessed weekly.

### 2.5. Tumor-Bearing Mouse Drug and Irradiation Treatment

The following equation was used to calculate tumor volume: volume = ab^2^/2 where *a* indicates the length and *b* indicates the diameter. When the tumor volume reached 100-200 mm^3^, tumor-bearing mice of the Cur (100 mg/kg curcumin dissolved in corn oil containing 2% DMSO), model (corn oil contained 2% DMSO), IR (corn oil containing 2% DMSO), and Cur+IR (100 mg/kg curcumin dissolved in corn oil containing 2% DMSO) groups were treated via intraperitoneal injections administered every other day. The Glu-GNPs+IR (4 mg/kg Glu-GNP suspension) group was treated via caudal vein injection weekly, and the Cis group (3 mg/kg cisplatin dissolved in normal saline) received an intraperitoneal injection every 3 days. Finally, the Cur+Glu-GNPs+IR group received 100 mg/kg curcumin via intraperitoneal injection every other day and 4 mg/kg Glu-GNPs via weekly caudal vein injections. Treatment lasted for a total of 3 weeks. The tumor-bearing mice of the IR, Glu-GNPs+IR, and Cur+Glu-GNPs+IR groups were subsequently treated with X-ray irradiation (10 Gray) using an X-Ray Irradiation Cabinet (MultiRad 225; Faxitron Bioptics, LLC). The tumor-bearing mice were euthanized when the nude mice were emaciated with vertebrae distinctly segmented to protect the welfare of nude mice based on the University of Pennsylvania IACUC guidelines. At the end of treatment, mice were euthanized by an intraperitoneal injection of 150 mg/kg 1% pentobarbital. All tumor tissue was extracted from tumor-bearing mice and weighed on the 24th day of treatment. A section of the tumor tissue was fixed by soaking in 4% polyformaldehyde (Biosharp Life Sciences) solution at 4°C for subsequent HE staining and immunohistochemistry. The other section of the tumor was transferred into liquid nitrogen and stored in the refrigerator at -80°C for subsequent reverse transcription-quantitative PCR detection. The synergistic effect between curcumin and Glu-GNPs was calculated using the following equation: *Q* = *E*_*a*+*b*_/(*E*_*a*_ + *E*_*b*_ − *E*_*a*_ × *E*_*b*_). *E*_*a*+*b*_ was the inhibition rate of the combined drugs, and *E*_*a*_/*E*_*b*_ was the inhibition rate of the single drug. *Q* < 0.85 means that drugs are antagonistic; *Q* ≥ 0.85 or *Q* < 1.15 means that drugs are additive, and *Q* ≥ 1.15 means that drugs exert a synergistic effect.

### 2.6. Tumor Growth Was Monitored Using an In Vivo Imaging System

After treatment for 24 days, the tumor-bearing mice were intraperitoneally injected with 150 mg/kg luciferase substrate (Catalog#luc001, Science Light, Co. Ltd., Shanghai, China) (http://sciencelight.biogo.net/) for *in vivo* imaging. Anesthesia was subsequently induced using 4% isoflurane in an oxygen-filled induction chamber. Once anesthetized, mice were placed in an imaging chamber and connected to the in-chamber anesthesia delivery system, which was maintained at 2% isoflurane. The bioluminescence intensity of all groups was detected and recorded within 10 min using the *in vivo* imaging system.

### 2.7. HE Staining

Tumor tissue samples were fixed in 4% paraformaldehyde at 4°C for 48 h. Samples were then dehydrated, embedded in paraffin, sectioned, and stained according to general procedure. The malignancy of tumor cells in each group was observed under a microscope (Carl Zeiss AG, Oberkochen, Germany) after sealing with neutral gum (Biosharp Life Sciences).

### 2.8. Immunohistochemical Analysis

VEGF (VEGF Rabbit monoclonal antibody; 1 : 50), HSP90 (HSP90 Rabbit monoclonal antibody; 1 : 50), HIF-1*α* (HIF-1*α* Rabbit monoclonal antibody; 1 : 500), and MMP9 (MMP9 Rabbit polyclonal antibody; 1 : 300) expressions were detected via immunohistochemistry. Tumor tissue samples were fixed in 4% polyformaldehyde solution, sliced with a paraffin-sectioning machine (Thermo Fisher Scientific, Inc.), stained using an immunohistochemistry kit (Bioss), dehydrated using a gradient series of ethanol solution, washed using xylene, and sealed with neutral gum. Positive cells were randomly selected from five fields of view and counted under an optical microscope (Carl Zeiss AG; magnification, ×400).

The IHC scoring method was as follows: 5 random fields of view were selected for each tissue. Briefly, the semiquantitative scoring was determined by the staining intensity and positive cell rate. The staining intensity was divided into four grades: 0 (negative), 1 (weak yellow), 2 (yellow), and 3 (tan). Positive cell rate dispersion method: 0 (<10%), 1 (11-25%), 2 (26-50%), 3 (51-75%), and 4 (>75%). And the total positive staining score was calculated as follows: staining intensity score × positive cell rate score.

### 2.9. RT-qPCR

Total RNA was extracted using TRIzol® (Thermo Fisher Scientific), and cDNA was synthesized using the Takara RNA Purification Kit according to the manufacturer's protocol. The amplification conditions were as follows: 95°C for 3 min, followed by 40 cycles of 95°C for 5 sec and 60°C for 30 sec. The experiment was concluded using the melt curve procedure. Relative expression was evaluated using the 2^-*ΔΔ*Cq^ method as the following: 2^−△△Cq^ = 2^[−(△Cq_control_△Cq_test_)]. RT-qPCR was performed using the following primers:

ACTB forward, 5′-GTGGCCGAGGATTTGATTG-3′ and reverse, 5′-CCTGTAACAACGCATCTCATATT-3′; GAPDH forward, 5′-AGCCACATCGCTCAGACAC-3′ and reverse, 5′-GCCCAATACGACCAAATCC-3′; VEGF forward, 5′-TAGAGTACATCTTCAAGCCGTC-3′ and reverse, 5′-CTTTCTTTGGTCTGCATTCACA-3′; HSP90 forward, 5′-ACGAAGCATAACGACGATGAGCAG-3′ and reverse, 5′-CCATTGGTTCACCTGTGTCAGTCC-3′.

### 2.10. Statistical Analysis

Statistical analyses were performed using a Student's *t*-test in Excel 2016 (Microsoft Corporation), SPSS 19.0 (IBM Corp.), and GraphPad Prism (GraphPad Software, Inc.). Comparisons between multiple groups were analyzed using ANOVA followed by Dunnett's multiple comparisons test. The results were presented as the mean ± standard deviation of triplicate experiments. *P* < 0.05 was considered to indicate a statistically significant difference.

## 3. Results

### 3.1. Cells Stably Express Firefly Luciferase

**Figure 1:**
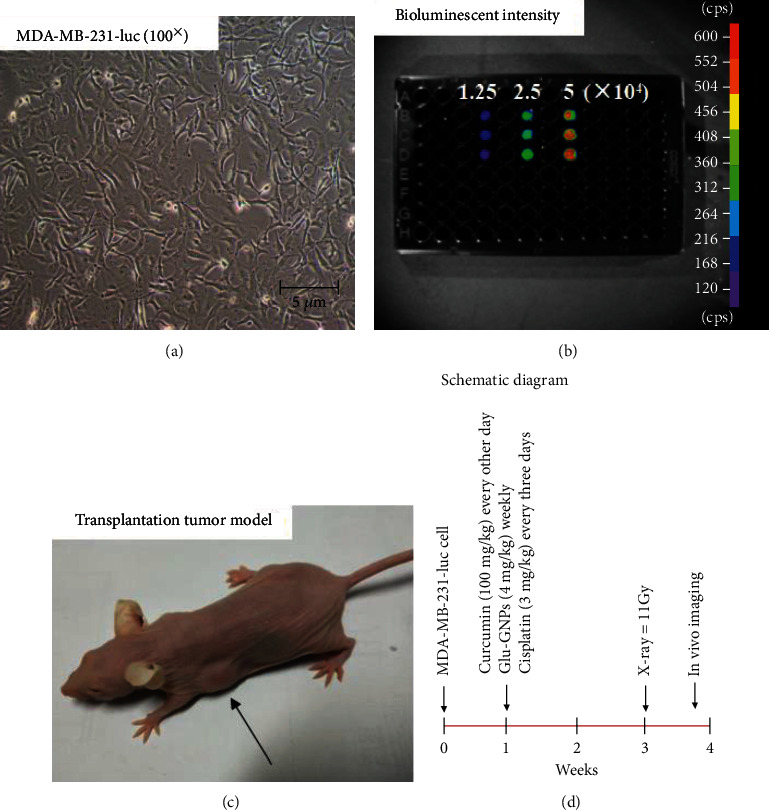
Cell culture and model establishment. (a) MDA-MB-231-luc cells were imaged (magnification, ×100; scale bar, 5 *μ*m). (b) The bioluminescence intensity of different numbers of cells (1.25 × 10^4^, 2.5 × 10^4^, and 5 × 10^4^) was detected using an *in vivo* imaging system in a black opaque 96-well plate. (c) A breast tumor-bearing mouse model exhibiting stable luciferase expression was successfully established 3-7 days after inoculation. (d) Schematic diagram of xenografts in nude mice. MDA-MB-231-luc cells were transplanted into the second pair of subcutaneous mammary glands on the left-hand side. When the tumor volume reached 100-200 mm^3^, curcumin in corn oil (2% DMSO), Glu-GNPs, and cisplatin in saline solution were administrated regularly. After 3 weeks of treatment, tumor-bearing mice (IR, Glu-GNPs+IR, Cur+IR, and Cur+Glu-GNPs+IR) were treated with irradiation (10 Gray). After further 3 days, the fluorescence intensity of mice in each group was detected using the *in vivo* imaging system. Glu-GNPs: glucose-gold nanoparticles; IR: irradiation; Cur: curcumin.

Bioluminescence was clearly observed in the cells of each well using an *in vivo* imaging system. As the number of cells increased, the bioluminescence intensity also increased (shown in Figures 1(a) and 1(b)). The results indicated that MDA-MB-231-luc cells could stably express firefly luciferase.

### 3.2. Transplanted Tumors Are Established Successfully in the Nude Mouse Model

At 3-7 days after inoculation, tumors were gradually formed in mice, with a tumor formation rate of 100%, excluding the control group (shown in Figure 1(c)). The tumors exhibited an almost sphere-like shape, with few appearing irregulars. Additionally, no significant change in body weight was demonstrated in the tumor-bearing mice. When the tumor volume reached 100-200 mm^3^, mice were regularly treated with different drugs for 3 weeks (shown in Figure 1(d)).

### 3.3. Tumor Growth Is Inhibited by Curcumin

**Figure 2:**
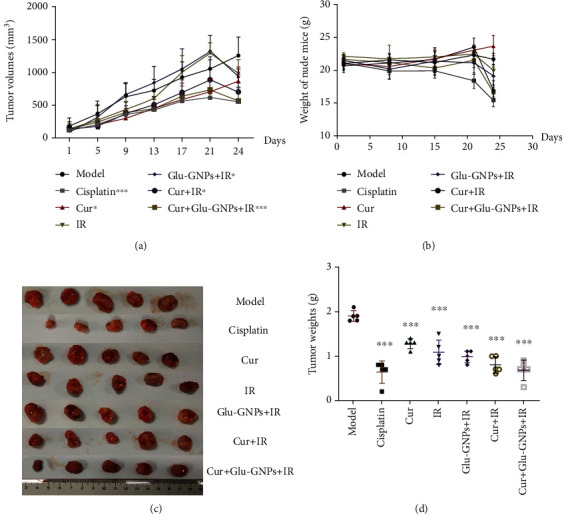
Tumor growth was decreased after treatment (*n* = 5). (a) Tumor volumes were measured every 4 days. (b) The weight of nude mice was measured weekly. (c) Images of transplanted tumor tissues which were removed after treatment and (d) weighed. Data were normalized to that of the model group, and data were presented as the mean ± SD. ^∗^*P* < 0.05, ^∗∗^*P* < 0.01, and ^∗∗∗^*P* < 0.001 vs. the model group (Student's *t*-test).

During treatment, tumor volume was measured every 3 days and the body weights of tumor-bearing mice were measured every other week. Cisplatin was used as positive control. The tumor volumes of the model group increased rapidly, while tumor growth in the other groups was slower, of which the cisplatin group grew the least quickly, also demonstrating the lowest tumor volume (*P* < 0.001). After X-ray irradiation, the tumor volumes of mice decreased significantly, particularly in the Cur+Glu-GNPs+IR group (shown in Figure 2(a)). Prior to radiotherapy, the body weights of mice did not markedly decrease, except for the Cis group. After 3 weeks of medication, the groups of tumor-bearing mice in IR, Cur+IR, Glu-GNPs+IR, and Cur+Glu-GNPs+IR received X-ray treatment. After irradiation, the body weights of the mice decreased significantly. Mice of the Cis group still exhibited weight loss. (shown in Figure 2(b)). The results demonstrated that curcumin inhibited tumor growth without obvious toxic side effects. The body weights of mice in the Cur+Glu-GNPs+IR group decreased significantly, indicating that X-ray irradiation had a negative impact on the survival of mice. After sampling, tumor tissue was weighed, and the tumor weight of each group differed statistically from the model group (shown in Figures 2(c) and 2(d)). The tumor weights of the Cis, Cur+IR, and Cur+Glu-GNPs+IR groups were reduced compared with model mice. The results indicated that curcumin administered with Glu-GNP and radiation served a combined therapeutic effect on breast cancer tumor growth.

### 3.4. Bioluminescence Intensity Detection Using the In Vivo Imaging System

**Figure 3:**
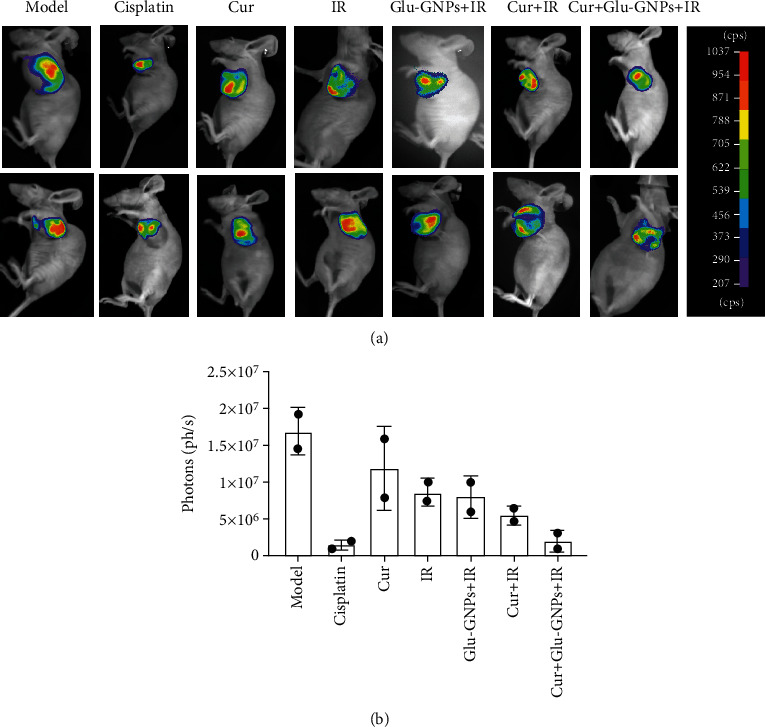
Bioluminescence intensity following *in vivo* imaging. (a) After treatment, the bioluminescence intensity of tumor-bearing mice that were intraperitoneally injected with luciferase substrate (150 mg/kg) was monitored using an *in vivo* imaging system (color). Mice were anesthetized by inhaling isoflurane. Bioluminescence was observed under the same conditions. (b) The bioluminescence intensity of each group was quantified.

The bioluminescence intensity of tumor growth was monitored 24 days after treatment using an *in vivo* imaging system. Two tumor-bearing mice of each group were randomly selected to detect their bioluminescence intensity. The results revealed that the bioluminescence intensity of the model group was the highest among all tumor-bearing mice. Furthermore, compared with the model group, the bioluminescence intensity of the Cis and Cur+Glu-GNPs+IR groups was significantly decreased (shown in Figures 3(a) and 3(b)). The results indicated that curcumin treatment inhibited tumor bioluminescence intensity and that an additive effect was demonstrated when curcumin was administered in combination with Glu-GNP and radiation.

### 3.5. Morphological Changes of Tumor Tissues after Treatment

**Figure 4:**
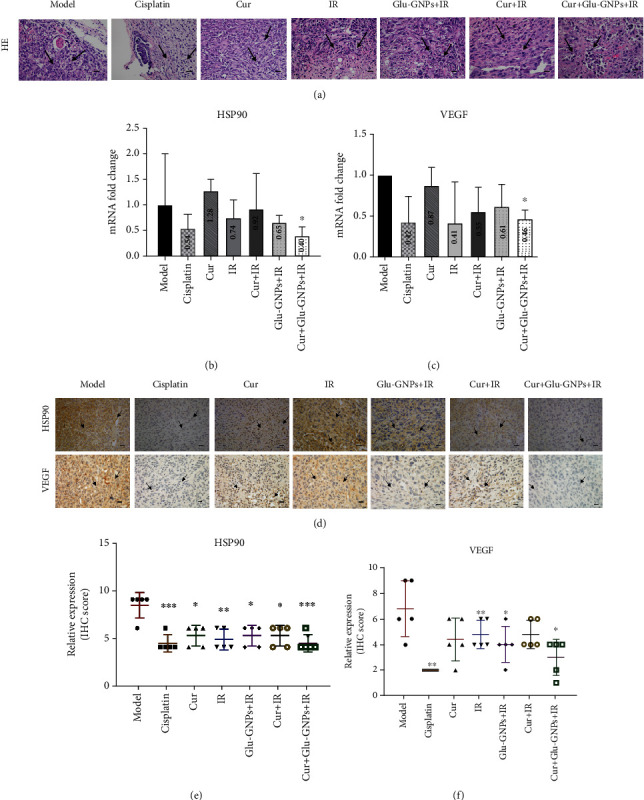
Detection of tumor tissue samples. (a) Representative images of HE staining (magnification, ×400) depicting the degree of malignancy in xenografts and tumor cells. The cells of tumor tissues exhibited large and dark nuclei with almost no cytoplasm and centrally located necrosis. Arrows indicate tumor cells (scale bars, 20 *μ*m). RT-qPCR analyses of (b) HSP90 and (c) VEGF mRNA levels in tumor tissue. *β*-Actin was used as a reference gene. Data were presented as the mean ± SD of three independent experiments. ^∗^*P* < 0.05 vs. the model group (Student's *t*-test). (d) Representative images of HSP90 and VEGF IHC staining (magnification, ×400). Tumor tissue was immunostained using DAB (brown) and hematoxylin (blue) for nuclear counterstaining. Arrows indicate the nuclear expression of HSP90 or VEGF in breast cancer cells (scale bars, 20 *μ*m). (e) The semiquantitative scoring analysis of HSP90 and (f) VEGF protein expression in tumor tissue. HE: hematoxylin and eosin; HSP90: heat shock protein 90.

After HE staining, the morphology of tumor tissue sections (4 *μ*m) was observed in each group under a microscope. The nucleus of tumor cells was large and heterogenous, with almost no cytoplasm and central necrosis. The results revealed that breast carcinoma xenografts were invasive ductal carcinomas with a high malignancy (shown in Figure 4(a)).

### 3.6. mRNA Levels Are Regulated following Treatment

The mRNA levels of VEGF and HSP90 were examined via RT-qPCR. When compared with the model group, mRNA levels of VEGF (Cis, ~0.42-fold; Cur, ~0.87-fold; IR, ~0.87-fold; Cur+IR, ~0.55-fold; Glu-GNPs+IR, ~0.61-fold; Cur+Glu-GNPs+IR, ~0.46-fold) and HSP90 (cisplatin, ~0.54-fold; Cur, ~1.28-fold; IR, ~0.74-fold; Cur+IR, ~0.92-fold; Glu-GNPs+IR, ~0.65-fold; Cur+Glu-GNPs+IR, ~0.40-fold) were regulated. The results demonstrated that curcumin and radiation significantly downregulated the expression of VEGF in tumor tissue, particularly in the Cur+Glu-GNPs+IR group (*P* < 0.05). When curcumin was administered in combination with Glu-GNP and radiation, the expression of HSP90 decreased significantly compared with the model group (*P* < 0.05; shown in Figures 4(b) and 4(c)). The results suggested that curcumin in combination with Glu-GNPs and radiation may be associated with the effective inhibition of VEGF and HSP90 in breast tumor tissue.

### 3.7. Immunohistochemical Analysis of the VEGF and HSP90 Genes

Five fields of view were randomly selected under microscopy, where brown staining indicated positive expression. The expression of VEGF decreased in the Cur, Cis, and Cur+Glu-GNPs+IR groups. HSP90 expression in the IR group was higher than that of the model group but decreased after curcumin treatment (shown in Figures 4(d)–4(f)). Radiation promoted the expression of HSP90, while curcumin administered with irradiation downregulated the levels of HSP90, protecting normal cells and inhibiting the growth of tumor cells. The results demonstrated that curcumin inhibited the growth of tumor vessels and exhibited a radiosensitization effect when administered alongside Glu-GNPs and X-ray therapy.

### 3.8. Immunohistochemical Analysis of the MMP9 and HIF-1*α* Genes

**Figure 5:**
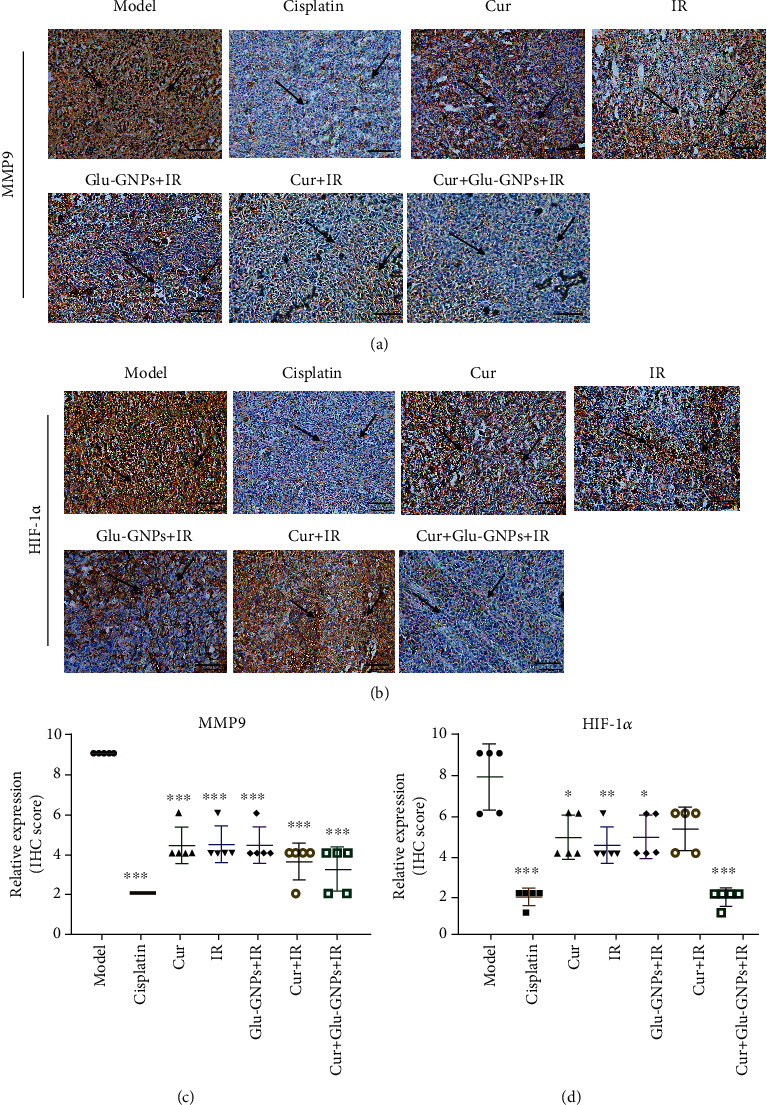
The protein expressions of MMP9 and HIF-1*α* in tumor tissue samples by immunohistochemistry. (a) Representative images of MMP9 and (b) HIF-1*α* IHC staining (magnification, ×200). Tumor tissue was immunostained using DAB (brown) and hematoxylin (blue) for nuclear counterstaining. Arrows indicate the nuclear expression of MMP9 or HIF-1*α* in breast cancer cells (scale bars, 100 *μ*m). (c) The semiquantitative scoring analysis of MMP9 and (d) HIF-1*α* protein expression in tumor tissue. IHC: immunohistochemistry; MMP9: matrix metalloprotein-9; HIF-1*α*: hypoxia-inducible factor-1*α*.

Studies confirm that radioresistance is closely related to angiogenesis [21]. It is well known that hypoxic in tumor microenvironment by HIF-1*α* activation can promote angiogenesis, tumor growth, metastasis, and cancer resistance [22]. Curcumin has been found to have antioxidant and antitumor angiogenesis effects in recent years. We wanted to further study the effect of curcumin and Glu-GNPs administered with X-ray irradiation on angiogenesis and radioresistance in breast cancer. Therefore, we detected the expression of genes HIF-1*α* and MMP9 related to angiogenesis. The results demonstrated that the expression of MMP9 and HIF-1*α* in the model group was higher than that in other groups, and the expression of MMP9 and HIF-1*α* decreased after curcumin, IR, and Glu-GNP treatment, respectively. Furthermore, we found that when curcumin and Glu-GNPs administered with X-ray irradiation, the expression of MMP9 and HIF-1*α* downregulated significantly compared with other treatment groups except for the cisplatin group (Figures 5(a)–5(d)). These results suggested that curcumin and Glu-GNPs administered with X-ray irradiation had an additive therapeutic effect.

## 4. Discussion and Conclusion

Curcumin is a plant polyphenol with effective activities such as anticancer and anti-inflammatory that is used in everyday foodstuffs. Previous studies [23] have reported that curcumin demonstrates anticancer activity, low toxicity, and reduced side effects. A previous study [24] also determined that curcumin effectively inhibited MDA-MB-231 and MCF-7 cells *in vitro*. Furthermore, curcumin administered in combination with a variety of anticancer drugs has been determined to enhance the sensitivity of cancer cells, which has gained increasing interest [25]. Previous research [26] has demonstrated that curcumin and Glu-GNPs, administered alone and in combination, inhibited proliferation and clone-forming on adherent cells and their mammospheres following X-ray irradiation, indicating that Glu-GNPs may be a promising radiosensitizer of MDA-MB-231 adherent cells and stem cells. However, the influence of curcumin on the radiosensitivity of breast carcinoma *in vivo* and its combined effect with Glu-GNPs remains unclear. Roa et al. used different doses of thiol-6-FDG-GNPs (35, 50, 67.6, 84.5, 101.5, and 120 mg Au kg^−1^) to evaluate the toxicity *in vivo*, and results showed that there was no acute toxicity in all doses during one-week observation [27]. In this study, we selected a dose of 4 mg/kg Glu-GNPs, far lower than the maximum tolerated dose.

The present study selected the luciferase-labeled cell line, MDA-MB-231-luc, and established a transplanted tumor model following the subcutaneous inoculation of cells into the underarms of mice, with a tumor formation rate of 100%. Nude mice were treated for 3 weeks with cisplatin as a positive control. According to the tumor volumes and body weights of mice, the curative effect of drugs was preliminarily evaluated. The results demonstrated that cisplatin was an effective treatment. However, marked weight loss was observed in the Cis group. Curcumin-treated groups demonstrated inhibited tumor growth without significantly affecting body weight.

The bioluminescence intensity of tumor-bearing mice in each group was measured using an *in vivo* imaging assay. The results demonstrated that the tumor bioluminescence intensity of the model group was highest among the treatment groups, while Cur, Cur+IR, and Cur+Glu-GNPs groups demonstrated a weaker bioluminescence intensity. This decrease was the most marked in mice of the Cur+Glu-GNPs+IR and Cis groups. Therefore, it could be concluded that curcumin inhibited the growth rate of transplanted tumors and demonstrated a combined therapeutic effect when administered with Glu-GNPs and X-ray.

Studies have demonstrated that tumors are able to resist radiotherapy as their blood vessels are irregularly distributed, meaning that blood circulation is blocked, which results in hypoxic areas of tumor tissue [28]. Hypoxic cells demonstrate high radiation tolerance and resist tumor stem cell DNA damage [29]. Angiogenesis is a marker of tumor growth, and VEGF plays a pivotal role in angiogenesis [30]. VEGF is considered to be one of the most critical factors involved in angiogenesis, and it is highly expressed in tumor tissues. VEGF, activated by binding with VEGFR, promotes the proliferation and migration of tumor cells and inhibits the apoptosis of tumor cells. Studies showed that the prognosis of cancer patients with VEGF positive is worse than that with VEGF negative [31]. The results of the current study revealed that curcumin administered with Glu-GNPs significantly reduced VEGF mRNA and protein levels (*P* < 0.05). This treatment also reduced HSP90 production in tumor tissue. VEGF is a dimeric glycoprotein secreted by several types of cells, including cancer and peripheral blood mononuclear cells. It is an angiogenesis factor that serves an important role in tumor growth in mice and is a poor prognostic indicator for different types of cancer [32]. HSP90 is a type of stress protein, the levels of which can rise sharply and promote tumor growth when stimulated by the environment [33]. HSP90 is considered to be a “cancer chaperone” as its presence is necessary to maintain the stability, function, apoptosis, and self-renewal of cancer cells [34]. The present study revealed that the expression of VEGF and HSP90 decreased following curcumin treatment, particularly when curcumin was administered in combination with Glu-GNPs and irradiation. Furthermore, when compared with the model group, HSP90 and VEGF levels and tumor volumes decreased significantly. This indicated that the therapeutic effect of curcumin, Glu-GNPs, and irradiation may be associated with the inhibited expression of HSP90 and VEGF in tumor-bearing mice. To further study the effect of curcumin and Glu-GNPs administered with X-ray irradiation on angiogenesis and radioresistance in breast cancer, we further detected the expression of genes HIF-1*α* and MMP9 related to angiogenesis. The results demonstrated that the expression of MMP9 and HIF-1*α* in the Cur+Glu-GNPs+IR group downregulated significantly compared with other treatment groups and model groups. The results further confirmed that curcumin and Glu-GNPs administered with X-ray irradiation can inhibit angiogenesis, thus inducing radiosensitivity.

Our previous *in vitro* studies revealed that MCF-7 and MDA-MB-231 breast cancer cells absorbed more Glu-GNPs compared with GNPs. Glu-GNPs are mainly absorbed by cancer tissue since tumor cells can take in more glucose. Therefore, the killing effects of these nanometal particles were enhanced but did not increase damage to the surrounding normal tissue in a mouse model. The effects of radiotherapy were therefore reduced, indicating that Glu-GNP could serve as a potential radiosensitizer. Moreover, curcumin is the main ingredient of curry, with the characteristics of high efficiency, safety, and low toxicity. Our study demonstrated that curcumin inhibited tumor growth without obvious toxic side effects. However, the body weights of the mice in the cisplatin group were significantly decreased *in vivo*. The combination of curcumin and Glu-GNPs demonstrated a good combined effect in breast cancer *in vivo*. In this study, we used intravenous injection and intraperitoneal injection, respectively. Nowadays, some studies used a liposome nanodrug codelivery system to combine multianticancer drugs to achieve the synergistic anticancer effect, overcome the drug resistance, and reduce the side effects [35]. In addition, a study found that a polymer-based drug codelivery system could enhance and accelerate cellular uptake and reverse multidrug resistance [36]. At present, we are considering assessing curcumin structure modification or liposome encapsulation in future research. The combination of curcumin and Glu-GNPs by the codelivery system may be a potential future research direction.

## Data Availability

The data used to support the findings of this study are included within this article.

## Abbreviations

Cis: Cisplatin
Cur: Curcumin
Cur+Glu-GNPs: Curcumin+Glu-GNP group
Cur+Glu-GNPs+IR: Curcumin+Glu-GNPs+irradiation group
GNPs: Gold nanoparticles
Glu-GNPs: Glucose-gold nanoparticles
HE: Hematoxylin and eosin
IR: Irradiation.
